# Risk analysis of grade ≥ 2 radiation pneumonitis based on radiotherapy timeline in stage III/IV non-small cell lung cancer treated with volumetric modulated arc therapy: a retrospective study

**DOI:** 10.1186/s12890-022-02211-8

**Published:** 2022-11-07

**Authors:** Songhua Yang, Shixiong Huang, Xu Ye, Kun Xiong, Biao Zeng, Yingrui Shi

**Affiliations:** 1 Department of Clinical Pharmaceutical Research Institution, Hunan Cancer Hospital/the Affiliated Cancer Hospital of Xiangya School of Medicine, Central South University, Changsha, 410013 Hunan People’s Republic of China; 2 Department of Radiation Oncology, Hunan Cancer Hospital/the Affiliated Cancer Hospital of Xiangya School of Medicine, Central South University, Changsha, 410013 Hunan People’s Republic of China; 3 Department of Anatomy and Neurobiology, School of Basic Medical Science, Central South University, Morphological Sciences Building, 172 Tongzi Po Road, Changsha, 410013 Hunan China

**Keywords:** Radiation pneumonitis, Radiotherapy timeline, Nomogram, Risk classification system

## Abstract

**Background:**

Radiotherapy is an important treatment for patients with stage III/IV non-small cell lung cancer (NSCLC), and due to its high incidence of radiation pneumonitis, it is essential to identify high-risk people as early as possible. The present work investigates the value of the application of different phase data throughout the radiotherapy process in analyzing risk of grade ≥ 2 radiation pneumonitis in stage III/IV NSCLC. Furthermore, the phase data fusion was gradually performed with the radiotherapy timeline to develop a risk assessment model.

**Methods:**

This study retrospectively collected data from 91 stage III/IV NSCLC cases treated with Volumetric modulated arc therapy (VMAT). Patient data were collected according to the radiotherapy timeline for four phases: clinical characteristics, radiomics features, radiation dosimetry parameters, and hematological indexes during treatment. Risk assessment models for single-phase and stepwise fusion phases were established according to logistic regression. In addition, a nomogram of the final fusion phase model and risk classification system was generated. Receiver operating characteristic (ROC), decision curve, and calibration curve analysis were conducted to internally validate the nomogram to analyze its discrimination.

**Results:**

Smoking status, PTV and lung radiomics feature, lung and esophageal dosimetry parameters, and platelets at the third week of radiotherapy were independent risk factors for the four single-phase models. The ROC result analysis of the risk assessment models created by stepwise phase fusion were: (area under curve [AUC]: 0.67,95% confidence interval [CI]: 0.52–0.81), (AUC: 0.82,95%CI: 0.70–0.94), (AUC: 0.90,95%CI: 0.80–1.00), and (AUC:0.90,95%CI: 0.80–1.00), respectively. The nomogram based on the final fusion phase model was validated using calibration curve analysis and decision curve analysis, demonstrating good consistency and clinical utility. The nomogram-based risk classification system could correctly classify cases into three diverse risk groups: low-(ratio:3.6%; 0 < score < 135), intermediate-(ratio:30.7%, 135 < score < 160) and high-risk group (ratio:80.0%, score > 160).

**Conclusions:**

In our study, the risk assessment model makes it easy for physicians to assess the risk of grade ≥ 2 radiation pneumonitis at various phases in the radiotherapy process, and the risk classification system and nomogram identify the patient’s risk level after completion of radiation therapy.

## Background

Non-small-cell lung cancer (NSCLC) accounts for 85% of all lung cancer cases [1]. Many NSCLC cases were diagnosed as stage III/IV and lost the opportunity for surgery, making it a major cause of cancer-associated death globally [2]. Therefore, systemic therapy was the main therapeutic modality for stage III/IV NSCLC, with radiotherapy playing an important role. Radiation pneumonitis (RP) was the most common and potentially devastating side effect of thoracic radiotherapy, occurring within 6 months post-RT. It caused chronic respiratory insufficiency, even severely affect patients’ life quality, caused the dismal prognostic outcome, and even death [3–5]. Patients with III/IV NSCLC have the highest risk of RP, with data from studies reporting up to 30 to 40% [6]. Therefore, it is essential among III/IV NSCLC cases to identify toxicity in RT as soon as possible. Moreover, it is necessary to accurately assess the RP risk, allowing for personalized RT dosing and maximized therapeutic benefits.

Numerous studies have been conducted to investigate clinical risk factors for RP, such as pulmonary function, performance status, smoking history, tumor location, an interstitial pulmonary disorder, pulmonary emphysema, and concurrent chemotherapy, all of which are strongly associated with the occurrence of RP [6–11]. Previously, RP has been reported to be related to dosimetric factors derived from dose-volume histograms, such as average lung dose, V5, V20, or dosiomics parameters [12–15]. As radiomics analytical techniques rapidly develop, research on RT therapeutic benefits and estimating its adverse reactions according to radiomics characteristics have become the research hotspots [16–19]. As per an article, changes in certain radiomics characteristics were dose-dependently related to RP grade ≥ 2 determined by obtaining local lung CT images post-RT [18]. In addition, a study constructed the model to differentiate patients with high-risk RP from low-risk RP by analyzing the region of interest (ROI) within the entire lung tissue prior to RT [19]. Briefly, radiomics characteristics can be used to obtain lung texture characteristics and aid in describing possible RP risk [20, 21]. However, the vast majority of studies and analyses were based on one individual data type or a simple combination of several data types. In practice, the duration of the radiotherapy process can take up to 8–10 weeks and consists of four phases: admission examination, target delineation, plan design, and radiotherapy implementation. Different types of data can be available at different radiotherapy phases; therefore, a longitudinal analysis of radiotherapy data based on a timeline may be more meaningful.

The present work investigated the potential risk factors for RP at different phases of radiotherapy for stage III/IV NSCLC, then fused the data of different phases based on the radiotherapy timeline to improve the effectiveness of the model, and finally established the risk classification system and nomogram according to fusion model of all phases data. In the age of precision radiotherapy, these tools can help physicians identify patients with RP as early as possible, allowing them to customize follow-up treatment strategies and interventions.

## Materials and methods

### Patient cohort

The present retrospective study included 91 cases between June 2019 and June 2021. All patients were diagnosed with stage III/IV NSCLC according to the AJCC 8th edition. Inclusion criteria: (1) No contraindication to RT and estimated survival greater than 6 months after RT. (2) No intolerance or interruption of RT for more than a week. (3) Absence of acute infectious or autoimmune disease. (4) Complete follow-up information. The gross tumor volume (GTV), planning target volume (PTV), and clinical target volume (CTV) as defined in ICRU 50 and 62 were measured in this study. Furthermore, this study described risky organs and target volumes based on RT and oncologic group guidelines. The prescribed RT doses were 50–66 Gy at 1.8–2 Gy/fraction/day for five fractions/week. Each RT plan was obtained from the Eclipse system (Varian Medical Systems, Palo Alto, CA, version 13.5.35), which was delivered with 6 MV photon beams and 2-arc VMAT. Patients were given either simultaneous chemoradiation or sequential chemoradiation. The chemotherapy regimens contained Carboplatin-based doublet, Cisplatin-based doublet, Platinum-based triplet, Single agent and Other which were applied in a widespread manner inclinical settings. Doses and regimens were modified according to the National Comprehensive Cancer Network (NCCN) and the Chinese Society of Clinical Oncology (CSCO). Informed consent was not required as this is a retrospective, unicentric cohort study.

### Evaluation of RP

All cases were assessed every week during the process of RT. Follow-up visits were made 1 month after RT and every 2–3 months for the next 6 months. RP was graded by one senior radiologist and two senior oncologists. RP severity was assessed by the National Cancer Institute Common Terminology Criteria for Adverse Events 4.03 (CTCAE 4.03). A grade ≥ 2 was considered symptomatic RP, which required steroids or limiting instrumental activities of daily living. Therefore, the endpoint was Grade ≥ 2 RP (RP ≥ 2) in our study.

### Four-phase characterization based on radiotherapy timeline

**Fig. 1:**
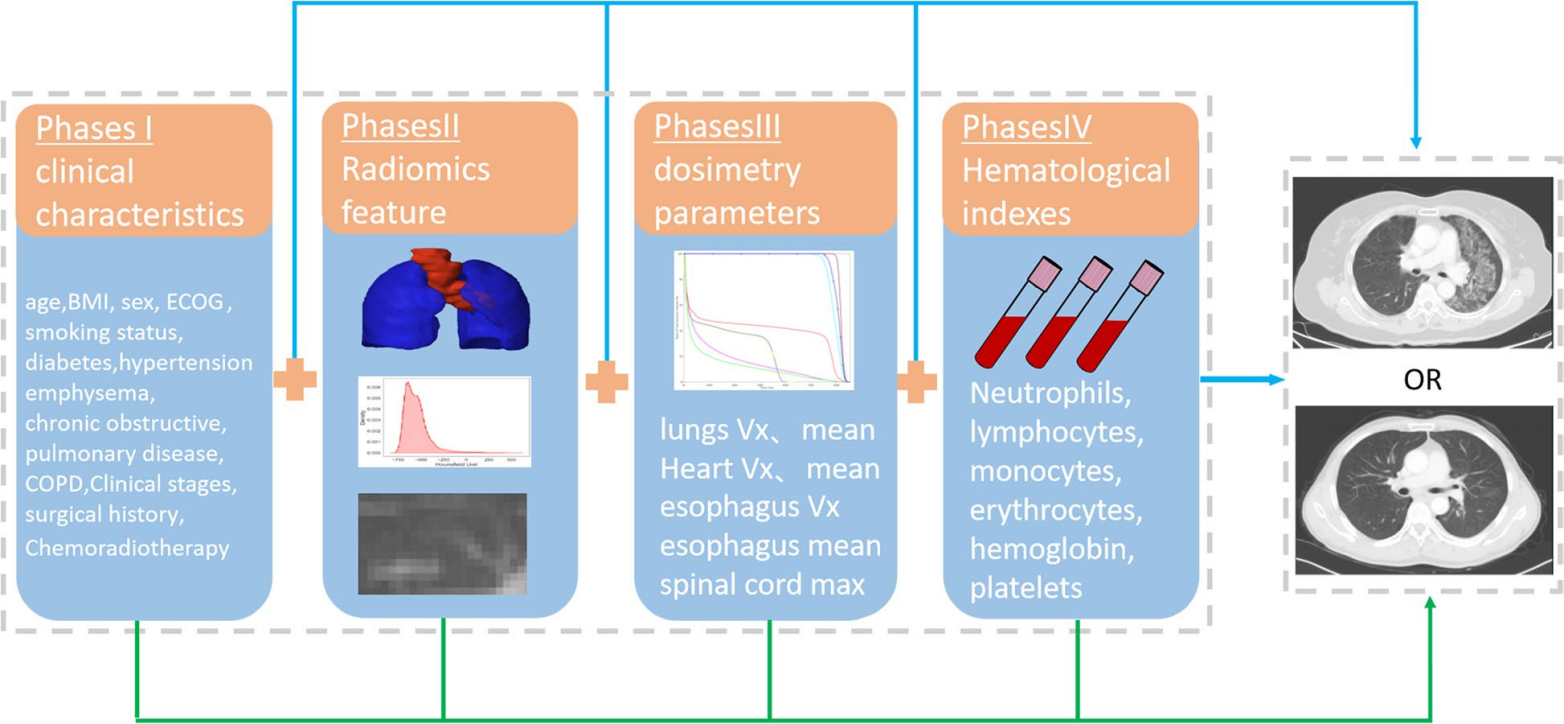
An overall workflow of risk analysis of RP ≥ 2

This study was used to investigate RP ≥ 2 risk by collecting data at four different phases, as shown in Fig. 1 (1) General clinical characteristics of patients at the admission examination phase (Phase I): age, BMI, sex, ECOG score, smoking status, diabetes, hypertension, emphysema, chronic obstructive pulmonary disease (COPD), Clinical stages, surgical history, and chemoradiation. (2) Radiomics feature at target area delineation phase (Phase II): Radiomics feature of PTV and lungs. (3) radiation dosimetry parameters during the RT plan design phase (Phase III): lungs V5-V50 (by 5), mean lung dose, Heart V5-V50 (by 10), mean heart dose, esophagus V5-V50 (by 10), mean esophagus dose, and maximum spinal cord dose. This study defined lung as (left+right lung)-GTV, whereas Vx as total lung volume that received xGy or higher level of radiation. (4) Hematological indexes of patients during the radiotherapy implementation phase (Phase IV): This study required neutrophils, lymphocytes, monocytes, erythrocytes, hemoglobin, and platelets at three distinct time intervals with 1 week before RT (baseline), 3 weeks (3w) during RT, and 5 weeks (5w) during RT.

### CT image acquisition and radiomics features extraction

All cases were subjected to free-breathing CT scans using a Philips Brilliance Big Bore CT scanner (Philips Medical Systems, Inc., Cleveland, OH) to develop treatment plans. The following parameters were used in CT scans: tube current (200 mA), voltage (120 kVp), pixel size (0.911 mm), slice thickness (5 mm), and image matrices (X: 768, Y: 768). This work utilized the Pyradiomics library in Python for extracting radiomic features. One hundred five Original features were extracted, including 18 first-order features, 14 shape features, and 73 texture analysis features (Gray Level Concurrence/Run Length/Size Zone/Dependence Matrix [GLCM/GLRLM/GLSZM/GLDM features, Neighborhood gray-tone difference matrix [NGTDM] features) were extracted. A total of 1183 transformation features based on shape and first order were extracted. Eight wavelet filters (LHL, HLL, LLH, LHH, HHH, HLH, LLL, and HHL) and five Image algorithms (square root, square, gradient, exponential, logarithm) were used. Prior to calculating radiomics features, each image was resampled (size: 3 × 3 × 3) and normalized (normalize Scale:100). We obtained 2576 features in PTV and lungs by performing the above operations.

### Statistical analysis

Univariate analysis was performed on Phase I/III/IV data. T-test and chi-square tests were used to calculate *P*-values for continuous and categorical variables, respectively. Subsequently, parameters of *P* < 0.05 were selected for multivariate analysis. This work utilized the least absolute shrinkage and selection operator (LASSO) algorithm for Phase II data for the sake of selecting significant features. Meanwhile, this work reduced coefficients of the unrelated radiomics feature to 0, while those (non-0) of the rest features closely associated with RP ≥ 2 were selected for multivariate analysis. The four Phases (I/II/III/IV) data were analyzed by multivariate analysis to establish the risk analysis model, and the significant risk factors of a single-phase model were included in the fusion phases (I ~ II/I ~ III/I ~ IV) model. The stepwise logistic regression was utilized in multivariate analysis. This work determined Spearman’s correlation coefficients in all models, with a result greater than 0.8 indicating potent correlation. For two closely related features, the feature with an larger univariate analysis *p*-value was removed [22, 23]. Variance Inflation Factor (VIF) is used to detect the presence of multicollinearity in model features. This work also plotted receiver operating characteristic (ROC) curves for evaluating model classification performance. Python and R software (version 3.5.3) was used for data analysis and visualization.

### Nomogram and risk classification system establishment and verification

This study developed a visual nomogram based on the fusion model of all phases data. As revealed by calibration curves (resampling of 1000 bootstraps), for RP ≥ 2, the nomogram-predicted probability was consistent with the actual value. Moreover, this work utilized the decision curve analysis (DCA) for testing nomogram utility in the clinic. The total risk point was assessed using our nomogram; then, recursive partitioning analysis was performed to develop the risk classification system for the accurate and effective classification of cases in diverse RP ≥ 2 risk levels.

## Results

### Univariate regression and radiomics feature selection

**Fig. 2:**
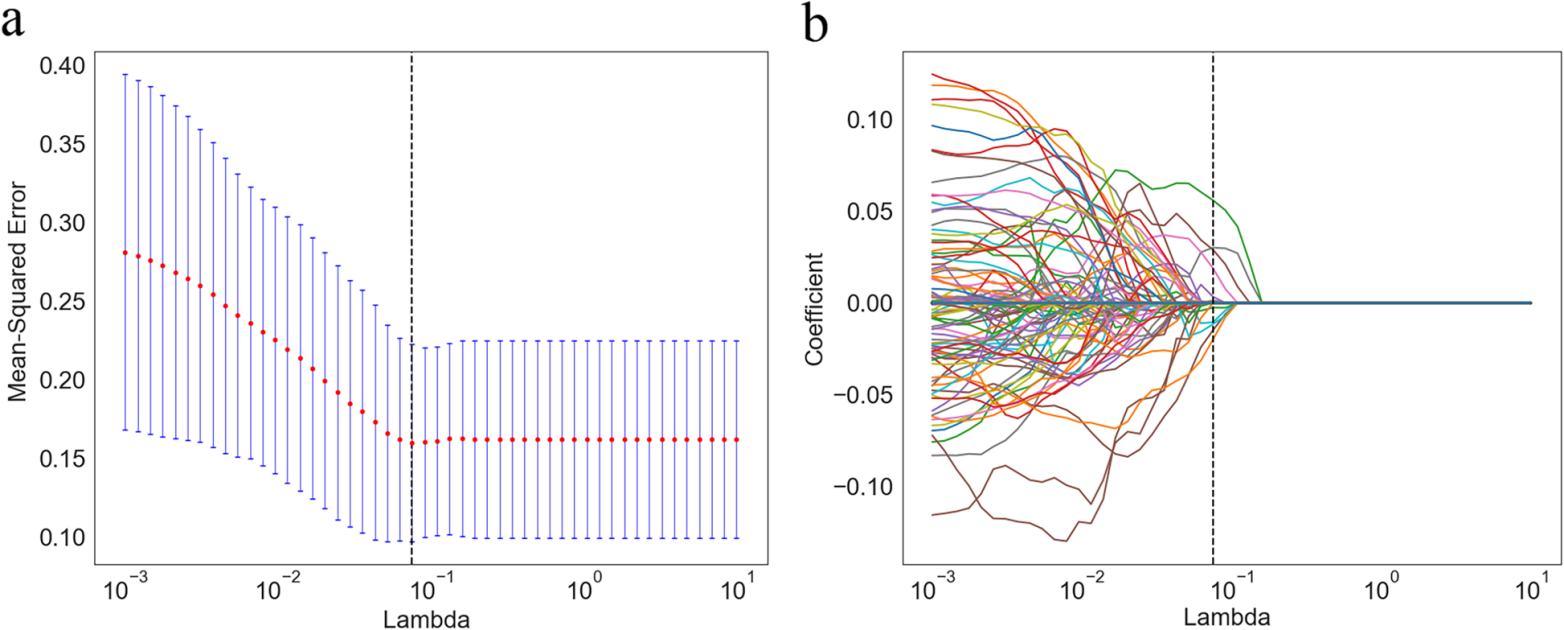
**a** Regulatory weight lambda screening. The best lambda value was defined by the vertical black dotted line. **b** LASSO coefficients for 2576 radiomics features. The best lambda value in (**a**) was defined by the vertical black dotted line, while ten features whose coefficients were non-0 were chosen at last. Abbreviation: LASSO = least absolute shrinkage and selection operator

The general clinical characteristics (Phase I) are shown in Table 1. In general, the incidence of RP ≥ 2 is 19.8% (18/91), of which sex (*P =* 0.01), BMI (*P =* 0.03), and smoking status (*P =* 0.004) showed significant differences of RP ≥ 2 when compared with RP < 2 groups. Lasso regression of Radiomics features (Phase II) is shown in Fig. 2. The feature selection results, which included PTV (2 shapes, 1 texture) and lung (5 first-order, 2 textures), are represented in Table 2. Radiation dosimetry parameters (Phase III) are shown in Table 3, where lungs v5–20 (by 5) and esophagus v10–20(by 10) are significantly different (*P* < 0.05) between the RP ≥ 2 group and RP < 2 group. Hematological indexes (Phase IV) are shown in Table 4. With the increase of RT weeks, the values of lymphocytes, erythrocytes, hemoglobin, and platelets show a decreasing trend. Among them, platelet in the third week (PLT_3W) of RT was significantly different (*P =* 0.011) between the groups.

**Table 1:** Univariate analysis results of clinical characteristics in Phases I

Clinical characteristics	RP < 2 (*n* = 73)	RP ≥ 2 (*n* = 18)	*p*-value
Age (year)	58.9 (8.8)	60.0 (6.5)	0.619
BMI	23.0 (2.5)	24.6 (2.8)	0.030*
Sex			0.010*
male	61 (85.92%)	10 (14.08%)	
female	12 (60.0%)	8 (40.0%)	
ECOG			0.279
0	19 (73.08%)	7 (26.92%)	
1	54 (83.08%)	11 (16.92%)	
Smoking Status			0.004**
yes	57 (87.69%)	8 (12.31%)	
No	16 (61.54%)	10 (38.46%)	
Diabetes			0.588
yes	7 (87.5%)	1 (12.5%)	
no	66 (79.52%)	17 (20.48%)	
Hypertension			0.342
yes	13 (72.22%)	5 (27.78%)	
no	60 (82.19%)	13 (17.81%)	
Emphysema			0.285
yes	11 (91.67%)	1 (8.33%)	
No	62 (78.48%)	17 (21.52%)	
COPD			0.704
yes	6 (85.71%)	1 (14.29%)	
no	67 (79.76%)	17 (20.24%)	
Clinical Stages			0.704
III	67 (79.76%)	17 (20.24%)	
IV	6 (85.71%)	1 (14.29%)	
Surgical History			0.666
yes	13 (76.47%)	4 (23.53%)	
no	60 (81.08%)	14 (18.92%)	
Chemoradiotherapy			0.871
concurrent	34 (80.95%)	8 (19.05%)	
sequential	39 (79.59%)	10 (20.41%)	

1. Data are Mean (standard deviation) or number of patients (percentages); 2.*P < 0.05, **P < 0.01

**Table 2:** Radiomics feature selection results of lasso regression in Phases II

Structure	Transformation	Type	Feature	Abbreviation
PTV	original	shape	MajorAxisLength	OSMAL_PTV
PTV	original	shape	Sphericity	OSS_PTV
PTV	exponential	glcm	Correlation	EGC_PTV
Lung	original	shape	Maximum2DDiameterRow	OSMax2DDR_Lung
Lung	logarithm	firstorder	Minimum	LFMin_lung
Lung	logarithm	firstorder	TotalEnergy	LFTE_Lung
Lung	exponential	firstorder	Maximum	EFMax_Lung
Lung	exponential	firstorder	MeanAbsoluteDeviation	EFMAD_Lung
Lung	exponential	glszm	SmallAreaLowGrayLevelEmphasis	EGSALG_Lung
Lung	exponential	ngtdm	Contrast	ENC_Lung

**Table 3:** Univariate regression results for dosimetry parameters in Phases III

Dosimetry parameters	RP < 2 (*n* = 73)	RP ≥ 2 (*n* = 18)	*p*-value
Lung			
V5(%)	48.14 (7.44)	54.58 (6.91)	0.001**
V10(%)	35.93 (5.96)	40.79 (5.25)	0.002**
V15(%)	28.99 (5.31)	32.96 (4.66)	0.004**
V20(%)	23.56 (4.74)	26.25 (3.81)	0.028*
V25(%)	19.38 (4.68)	21.08 (3.69)	0.157
V30(%)	16.07 (4.69)	17.17 (3.79)	0.355
V35(%)	13.19 (4.61)	13.96 (3.75)	0.514
V40(%)	10.69 (4.41)	11.1 (3.56)	0.718
V45(%)	08.47 (4.06)	8.68 (3.18)	0.841
V50(%)	6.43 (3.65)	6.39 (2.79)	0.957
Mean (GY)	12.96 (2.57)	14.21 (1.94)	0.056
Heart			
V10(%)	31.74 (14.98)	36.7 (20.41)	0.246
V20(%)	20.92 (11.12)	22.35 (16.36)	0.661
V30(%)	13.87 (8.58)	12.62 (11.23)	0.605
V40(%)	8.48 (6.28)	6.9 (7.81)	0.365
V50(%)	4.52 (4.1)	3.34 (3.99)	0.126
Mean (GY)	11.23 (5.18)	11.92 (6.62)	0.634
Esophagus			
V10(%)	49.2 (13.29)	60.39 (13.41)	0.002**
V20(%)	41.3 (14.44)	49.81 (16.77)	0.032*
V30(%)	35.09 (15.63)	39.72 (13.17)	0.250
V40(%)	28.42 (16.36)	32.67 (11.35)	0.204
V50(%)	20.84 (15.8)	24.24 (11.83)	0.395
Mean (GY)	21.71 (8.19)	25.23 (6.33)	0.092
SpinalCord			
max (GY)	38.73 (3.90)	38.29 (3.17)	0.277

1. Data are Mean (standard deviation); 2.*P < 0.05, **P < 0.01;3. Vx = percentage of structure volume receiving xGy

**Table 4:** Univariate regression results for Hematological indexes in Phases IV

Hematological indexes	RP < 2 (*n* = 73)	RP ≥ 2 (*n* = 18)	*p*-value
Neutrophils			
baseline (10^9/L)	3.94 (2.06)	3.76 (2.69)	0.202
3 W (10^9/L)	4.05 (4.73)	3.09 (2.01)	0.405
5 W (10^9/L)	5.23 (5.63)	4.81 (4.24)	0.881
△3 W	1.12 (0.99)	1.16 (1.01)	0.738
△5 W	2.15 (5.02)	2.01 (2.47)	0.488
Lymphocytes			
baseline (10^9/L)	1.79 (0.59)	1.93 (0.84)	0.393
3 W (10^9/L)	0.78 (0.35)	0.84 (0.41)	0.521
5 W (10^9/L)	0.57 (0.30)	0.56 (0.23)	0.914
△3 W	0.46 (0.19)	0.58 (0.67)	0.701
△5 W	0.34 (0.17)	0.38 (0.40)	0.621
Monocytes			
baseline (10^9/L)	0.60 (0.32)	0.59 (0.38)	0902
3 W (10^9/L)	0.59 (0.35)	0.55 (0.28)	0.612
5 W (10^9/L)	0.66 (0.38)	0.66 (0.30)	0.679
△3 W	1.25 (1.19)	1.77 (2.26)	0.900
△5 W	1.88 (4.03)	1.80 (1.49)	0.256
Erythrocytes			
baseline (10^12/L)	3.97 (0.63)	3.90 (0.75)	0.686
3 W (10^12/L)	3.80 (0.55)	3.69 (0.78)	0.463
5 W (10^12/L)	3.71 (0.66)	3.45 (0.84)	0.157
△3 W	0.98 (0.22)	0.94 (0.09)	0.607
△5 W	0.95 (0.24)	0.88 (0.15)	0.249
Hemoglobin			
baseline (g/L)	122.98 (15.49)	122.50 (18.19)	0.909
3 W (g/L)	117.54 (15.91)	116.61 (19.88)	0.833
5 W(g/L)	115.96 (19.99)	112.78 (22.69)	0.557
△3 W	0.96 (0.11)	0.95 (0.095)	0.778
△5 W	0.95 (0.14)	0.92 (0.14)	0.495
Platelets			
baseline (10^9/L)	213.36 (69.17)	209.61 (69.59)	0.837
3 W (10^9/L)	184.18 (52.25)	148.67 (48.55)	0.011*
5 W (10^9/L)	185.02 (69.93)	160.50 (88.12)	0.209
△3 W	0.91 (0.28)	0.78 (0.34)	0.097
△5 W	0.91 (0.32)	0.76 (0.27)	0.079

1. Data are Mean (standard deviation); 2. *P < 0.05;3. △3 W = the ratio of change at 3 weeks;△5 W = the ratio of change at 5 weeks)

### Multivariate analysis of single-phase data and fusion phases data

**Fig. 3:**
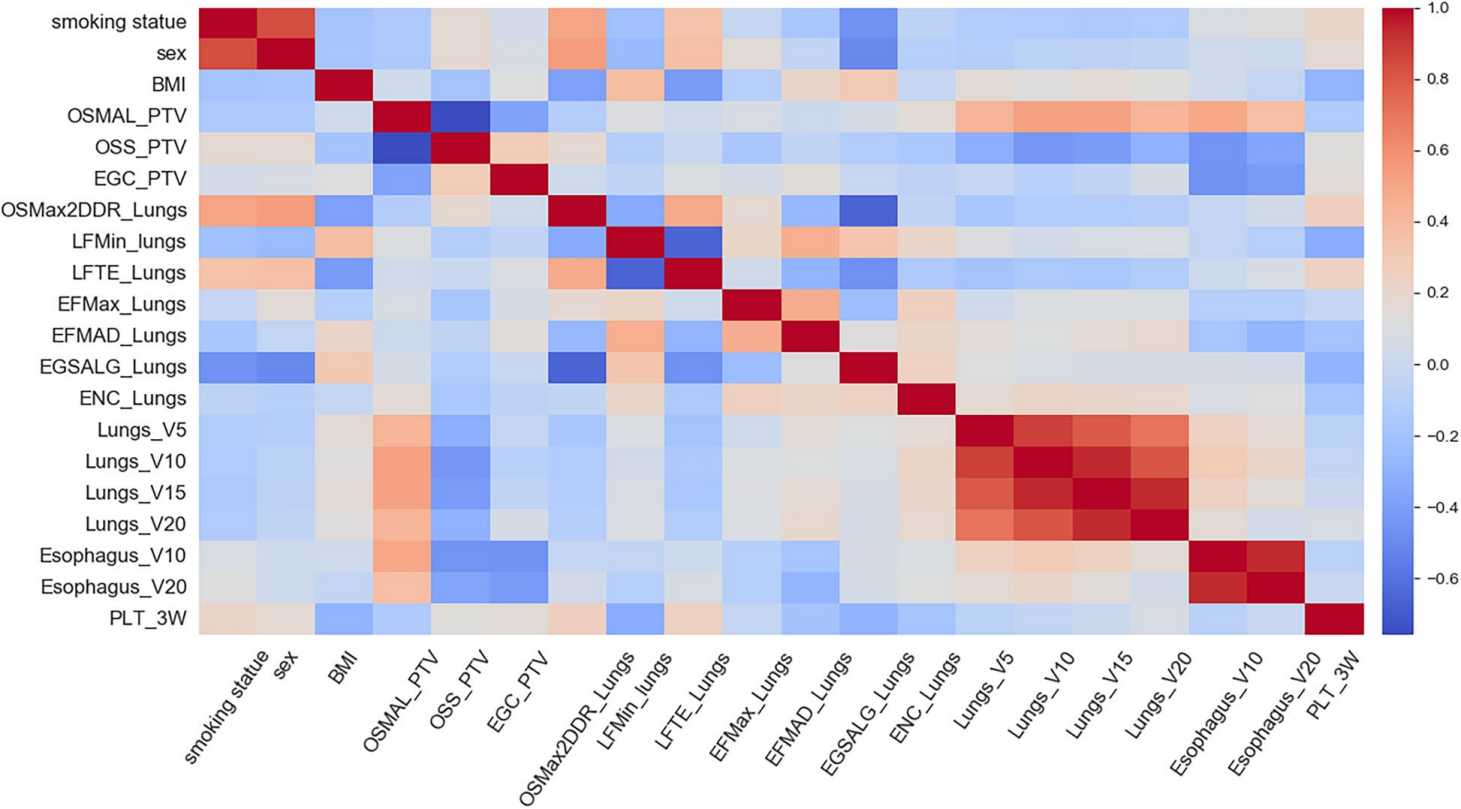
Feature Correlation Heat Map of univariate analysis results and lasso selection results. Heat map presenting the relation of features

**Fig. 4:**
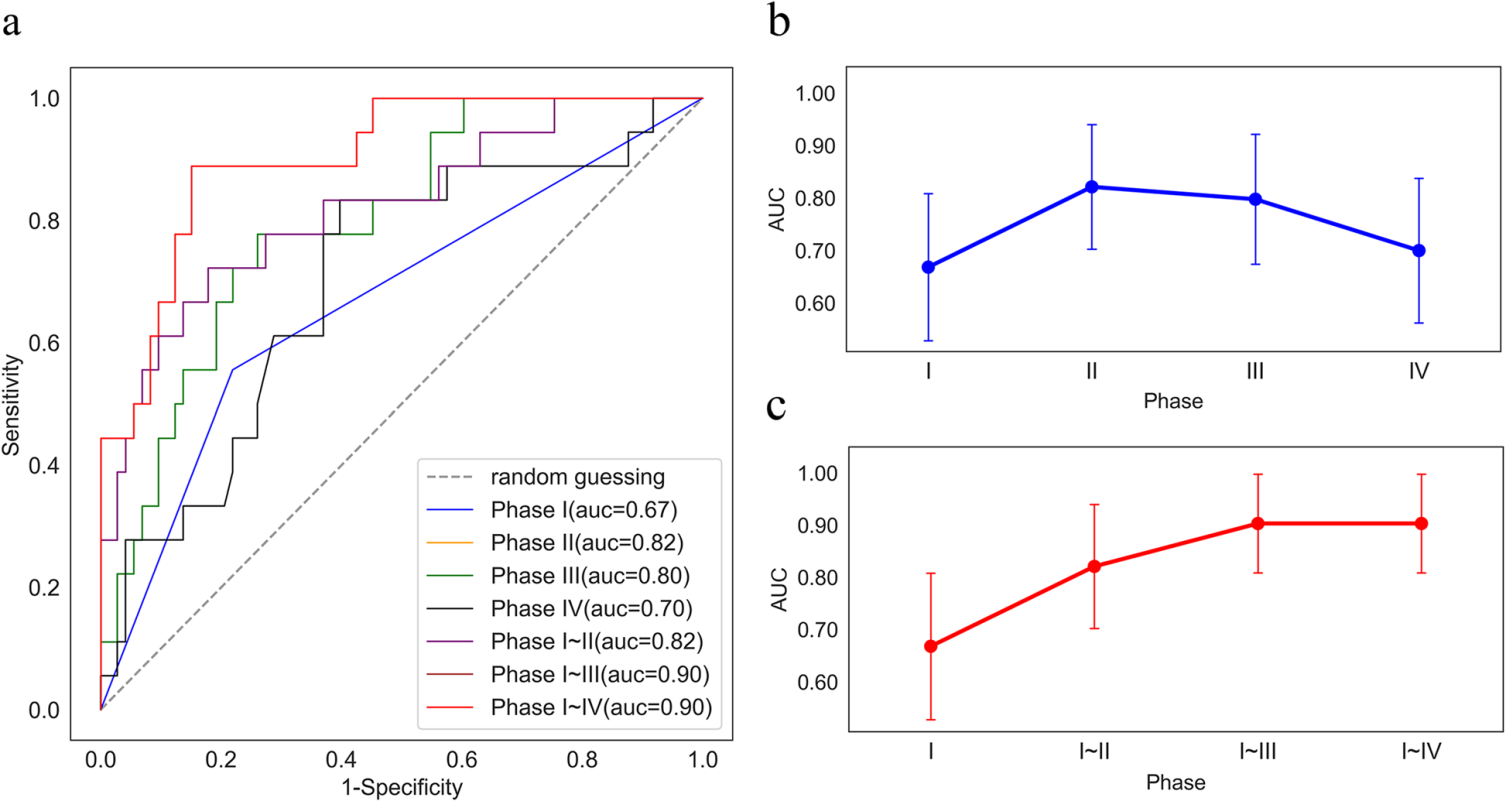
**a** Receiver operating characteristic curves (ROC) of single Phase and fusion Phase **b** Area Under Curve (AUC) trend curve of single Phase with timeline **c** AUC trend curve of fusion Phase with the timeline

The variable correlation coefficient after univariate analysis and feature selection is shown in Fig. 3. The multivariate analysis of single phases and fusion phases is depicted in Table 5. In the phase I model, smoking status is strongly correlated with sex (*r* > 0.8). Since smoking status (*P =* 0.004) was more significant than sex (*P* = 0.01) in the univariate analysis, sex was disregarded. The stepwise logistic regression result illustrated that smoking status was an independent risk factor (odds ratio [OR] = 0.27; 95% confidence interval [CI]: 0.08–0.85; *P* = 0.025). Similarly, OSMAL_PTV (OR = 1.02, 95% CI: 1.00–1.03, *p* = 0.018), LFMin_lung (OR = 1.01, 95%CI: 1.00–1.02, *P* = 0.003), Lungs_V5 (OR = 1.13, 95% CI: 1.03–1.23, *P* = 0.011), Esophagus_V10 (OR = 1.05, 95% CI: 1.00–1.11, P = 0.025), PLT_3W (OR = 0.98, 95% CI: 0.97–1, *P* = 0.014) were significant risk factors in Phase II/III/IV model. The independent risk factors of the four single phase models were fused by the RT timeline. Stepwise logistic regression was then performed for Phase I ~ II/I ~ III/I ~ IV models. Notably, the independent risk factors in the Phase I ~ II model comprised only two radiomics features without smoking status (OR = 0.3, 95% CI: 0.09–1.07, *P* = 0.064). Factors independently predicting RP risk in Phase I ~ III model involved clinical, radiomics, and dosimetry features. As PLT_3W (OR: 0.99, 95% CI: 0.97–1.00, *P* = 0.248) was eliminated from the Phase I ~ IV model, the components of independent factors were consistent with Phase I ~ III. The discriminatory ability of all model-independent risk factors was analyzed using ROC. The model area under curve (AUC), specificity, and sensitivity can be obtained from Table 6, and ROC visualization and AUC trends from Fig. 4. Notably, the AUC of Phase II (radiomics feature) model is highest in the single phase (AUC: 0.82, 95%CI: 0.70–0.94, Specificity: 0.82, Sensitivity: 0.72), followed by the Phase III (dosimetry parameters) model (AUC:0.80, 95%CI: 0.67–0.92, Specificity: 0.74, Sensitivity: 0.77). The AUC of the fusion model steadily increased with the radiotherapy timeline, and the results of the ROC analysis for the Phase I ~ III and Phase I ~ IV models are observed to be the same.

**Table 5:** Multivariable analysis results of single Phase and fusion Phase

	Coef	*P*-value	OR	VIF (max)
Phases I				1.06
Smoking (compared to non-smoking)	−1.2953	0.025*	0.27(0.08–0.85)	
BMI	0.1406	0.168	1.15(0.94–1.40)	
Phases II				1.12
OSMAL_PTV	0.0172	0.018*	1.02(1.00–1.03)	
LFMin_lung	0.0103	0.003**	1.01(1.00–1.02)	
EGC_PTV	−2.5103	0.060	0.08(0.00–1.12)	
Phases III				1.06
Lungs_V5(per 1%)	0.1189	0.011*	1.13(1.03–1.23)	
Esophagus_V10(per 1%)	0.0565	0.025*	1.05(1.00–1.11)	
Phases IV				1.00
PLT_3W	−0.0152	0.014*	0.98(0.97–1)	
Phases I ~ II				1.08
Smoking (compared to non-smoking)	−1.1966	0.064	0.30(0.09–1.07)	
OSMAL_PTV	0.0195	0.007**	1.02(1.00–1.03)	
LFMin_lung	0.0096	0.007**	1.01(1.00–1.02)	
Phases I ~ III				1.8
Smoking (compared to non-smoking)	−1.7608	0.021*	0.17(0.04–0.77)	
OSMAL_PTV	0.0031	0.741	1.00(0.98–1.02)	
LFMin_lung	0.0154	0.003**	1.01(1.00–1.02)	
Lungs_V5(per 1%)	0.1163	0.047*	1.12(1.00–1.26)	
Esophagus_V10(per 1%)	0.0977	0.009**	1.10(1.02–1.19)	
Phases I ~ IV				1.9
Smoking (compared to non-smoking)	−1.7533	0.025*	0.17(0.04–0.79)	
OSMAL_PTV	0.0028	0.769	1.00(0.98–1.02)	
LFMin_lung	0.0142	0.008**	1.01(1.00–1.02)	
Lungs_V5(per 1%)	0.1252	0.042*	1.13(1.00–1.27)	
Esophagus_V10(per 1%)	0.0950	0.010*	1.10(1.02–1.18)	
PLT_3W	−0.0091	0.248	0.99(0.97–1.00)	

1. Data are Mean (standard deviation);2. *P < 0.05, **P < 0.01

**Table 6:** ROC analysis results of single Phase and fusion Phase

Model	AUC(95% confidence interval)	Sensitivity	Specificity
Phase I	0.67(0.52–0.81)	0.56	0.78
Phase II	0.82(0.70–0.94)	0.72	0.82
Phase III	0.80(0.67–0.92)	0.77	0.74
Phase IV	0.70(0.55–0.84)	0.83	0.60
Phase I ~ II	0.82(0.70–0.94)	0.72	0.82
Phase I ~ III	0.90(0.80–1.00)	0.89	0.85
Phase I ~ IV	0.90(0.80–1.00)	0.89	0.85

### Nomogram and risk classification system

**Fig. 5:**
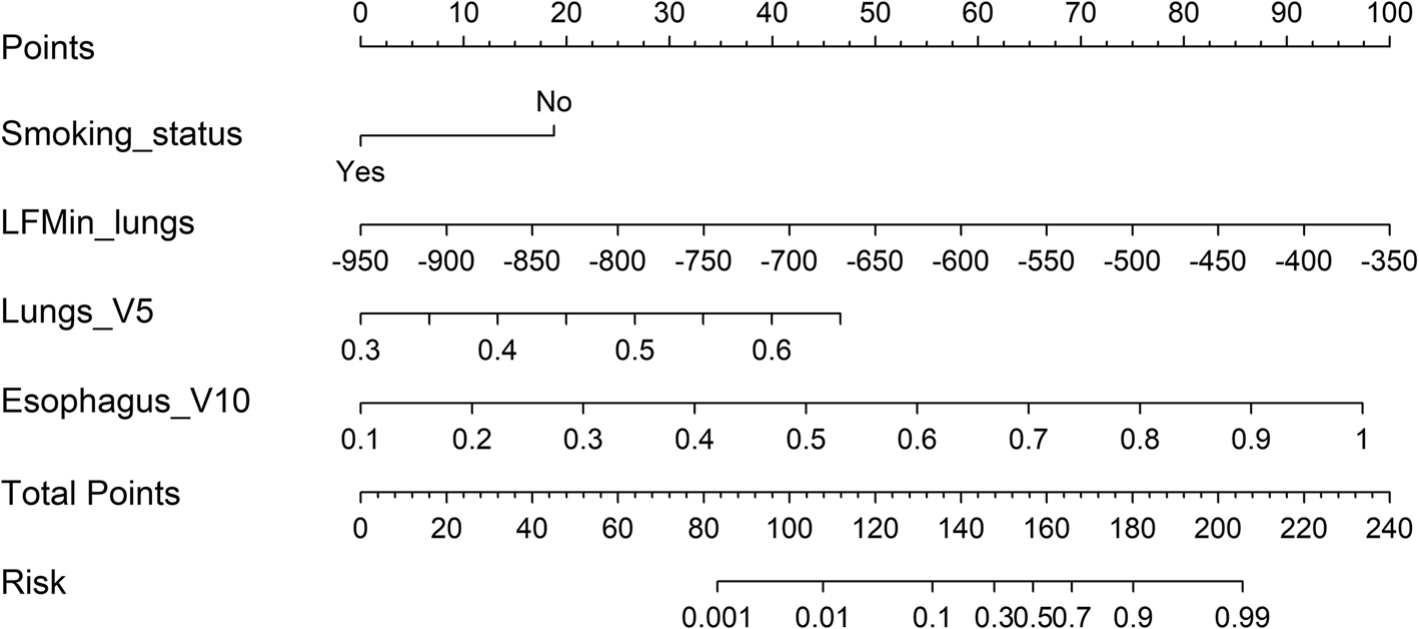
The nomogram incorporates Smoking status, LFMin_lungs, Lungs_V5, and Esophagus_V10 for analysing risk of RP ≥ 2

**Fig. 6:**
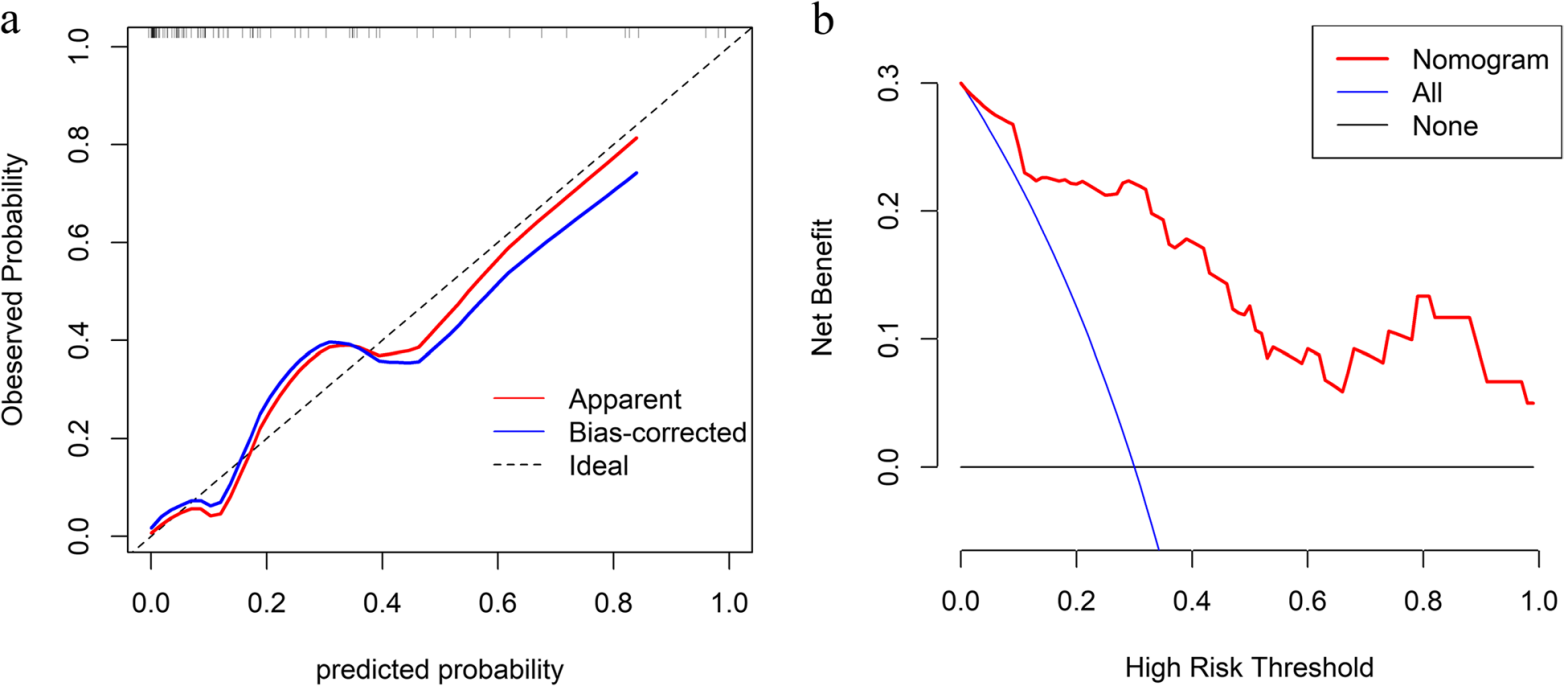
**a** Calibration curves for our constructed nomogram. An ideal assessment was represented by the diagonal dotted line, whereas our nomogram performance was indicated by the remaining two lines. **b** Decision curves for our constructed nomogram that analysed risk of RP ≥ 2. The y-axis stands for net benefit, whereas the red curve, horizontal black line, and oblique blue line stand for nomogram, valid and invalid assumption, separately

**Fig.7:**
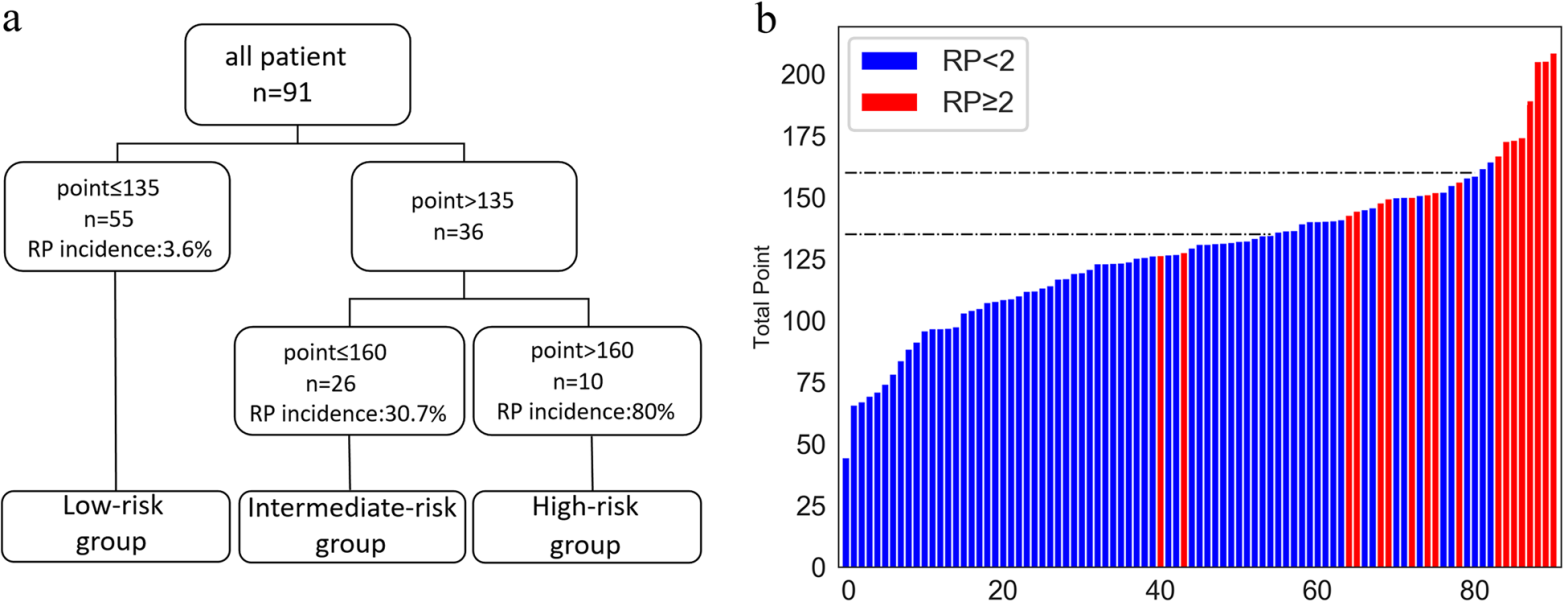
**a** The risk classification system. **b** Histogram of each patient’s nomogram point. All points were arranged in order. The red column represents RP ≥ 2 patients, the blue column represents RP < 2 patients, and the two dotted lines represent the threshold values of point for three different risk groups

Our nomogram based on Phase I ~ IV model is presented in Fig. 5. It included variables as follows: smoking status and LFMin_lung, lungs_V5, Esophagus_V10. The calibration curve verified well calibration of our constructed nomogram (Fig. 6a). DCA suggested that the risk analysis model might have good clinical benefits (Fig. 6b). The risk classification system, based on nomogram score, is shown in Fig. 7. The cases were classified as three diverse RP ≥ 2 risk levels: low-(score 0–135), intermediate-(135–160), or high-risk group (> 160), with the prevalence rates being 3.6% (2/55), 30.7% (8/26), and 80.0% (8/10), respectively. In this study, our established risk classification system was precise in differentiating cases having diverse RP ≥ 2 risk levels, therefore, facilitating decision-making in the clinic.

## Discussion

Smoking status was an independent risk factor in Phase I/I ~ III/I ~ IV models, which was supposed to be the protective factor for RP ≥ 2 (OR < 0.3, *P* < 0.05). In this study, the percentage of smokers or non-smokers with RP ≥ 2 was 12.31% (8/65) and 38.46% (10/26), respectively. These data suggested that smokers may be more tolerant to RT than non-smokers, which was in line with the related studies signifying the relation of smoking with a decreased hypersensitivity pneumonitis risk, possibly due to the immunosuppressive effect [24, 25]. However, that does not signify that patients are encouraged to smoke, as smoking affects lung cancer survival [26, 27]. It has been reported that there was no relationship between sex and RP ≥ 2 [8, 28]. The risk of RP ≥ 2 was 40% (8/20) in female and 14.08% (10/71) in male, depicting a higher risk in female when compared with male. Moreover, the difference was statistically significant (*P* < 0.05) and possibly associated with a much smaller female lung volume than the male lung volume (2055 ± 457 cm^3^ vs. 3207 ± 745 cm^3^, *P* < 0.001). Under similar dose and radiation field conditions, with the smaller lung volume and the larger the relative volume dose in female. However, sex was strongly correlated with smoking status (*r* > 0.8), with 0% (0/20) of women smoking and 91.5% (65/71) of men smoking. Sex was not involved in the multivariate analysis, therefore, further research was desired to determine the association of sex as an influential factor in RP ≥ 2.

The osmal_ptv, which was a PTV shape feature extracted from the target area by using the radiomics method, was an independent risk factor (or > 1.00, *P* < 0.05) of Phase II/I ~ II models that directly reflected the length information of the target area in the principal axis direction. Under similar conditions, the longer the target area, the larger the lung volume involved in RT, and the higher the risk of RP ≥ 2. However, the statistical significance of osmal_ptv for Phase I ~ III/1 ~ IV models was not significant (*P* > 0.05), possibly due to the introduction of the dosimetry parameters, particularly esophagus_V10 parameter. Presently, no literature has reported that esophagus dose was the direct influencing factor of RP. According to the anatomical relationship between the esophagus and lung, we predict that Esophagus_V10 can indirectly reflect the length information of the overlapping area of the target area and lung in the transverse position.

In many reports [29–31] that used radiomics to analyze RP risk, rad_signature was generally constructed based on the linear combination of non-zero coefficient parameters filtered by lasso regression. In this study, lasso selected ten features, but due to insufficient case data, especially the number of RP-positive patients (18), we avoided using the rad_signature method but used Spearman’s correlation coefficient and stepwise logistic regression to remove variables to avoid overfitting the model with too many variables. Currently, lung tissue radiosensitivity is suggested [32, 33] to be a potential influencing factor for RP. According to our results, LFMin_lung served as an independent risk factor (or > 1.00, *P* < 0.05) of Phase II/I ~ II//I ~ III/I ~ IV models. Therefore, we supposed that these radiomics feature extracted from lung CT images might express the RT sensitivity in lung tissues and differentiate susceptible groups with RP ≥ 2 that cannot be obtained from clinical characteristics or dosimetry parameters.

With the free-breathing mode used for the CT scanning modality in this study, it has been suggested that respiratory motion may simply be random noise that influences radiomic features and that features that predict symptomatic RP may be robust and reproducible features of the free-breathing protocol [34]. However, the effect of respiratory motion on radiomic features requires further study. In this paper the ROI of lung was obtained by semi-automatic way segmentation. The steps are as follows: first based on the Eclispe TPS automatic threshold segment tool, then using a manual segmentation method to erase the redundant parts of the lung beyond the large organs and lung parenchyma for the ROI. Image segmentation is performed by an experienced radiotherapist and then validated by a senior radiotherapist. It has been reported in the literature that manual segmentation is not only time consuming and can cause inter- and intra-observer errors, but semi-automatic segmentation is thought to increase stability [35, 36].

Studies have reported a close relationship between whole lung_V5 and RP in NSCLC [37–42]. Notably, lung_V5 was certainly significant for the prediction of RP among cases having mediastinal lymphoma and esophageal cancer receiving RT [43–45]. Based on the above results, lung_V5 possibly had a major impact on RP occurrence. As discovered in this work, lung_V5 was independent risk factor (or > 1, *P* < 0.05) in Phase III/I ~ III/1 ~ IV model. We also conducted a univariate ROC analysis on lung_V5. The best cut-off value was 52.7% (AUC = 0.74,95%CI:0.60–0.88, sensitivity = 0.78, specificity = 0.66). The reason why the cut-off value was only 52.7%, which was less than the dose parameter (v5 < 65%) of guidelines revealed by NCCN to reduce RP risk-limiting lung, might be that our institution has strict restrictions on lung_V5 in clinical practice (v5 < 60%).

PLT_3w was considered an independent risk factor (OR = 0.98, *P* = 0.01) in Phase IV model but not in Phase I ~ IV model (OR = 0.99, *p* = 0.248). However, it does not signify that platelets are not important because the difference was significant in RP ≥ 2 compared with RP < 2 groups upon univariate analysis (184.18 ± 52.25 10^^9^/L vs 148.67 ± 48.56 10^^9^/L, *P* = 0.011), with PLT_3W being lower in the RP ≥ 2 group. Similar results have also been reported in some studies [46] where platelets respond rapidly to resist pathogen invasion. Besides, they have an important effect on adaptive immunomodulation via T cells, B cells, and antigen-presenting cells (APCs). If the platelet number decreases compared to the normal level, patients will have poor immunity and will be more prone to RP. Some inflammatory biomarkers in serum, such as lactate dehydrogenase, C-reactive protein (CRP), tumor necrosis factor (TNF), interleukin, and transforming growth factor (TGF), are related to RP [47, 48]. However, in our study, based on ease of data acquisition, we collected only routine blood count data at different time points during the implementation of radiotherapy. Therefore, we might need further research in this area.

The lung cancer radiotherapy process typically includes admission examination, target area delineation, plan design, and radiotherapy implementation. The time required for the entire process slightly varied from hospital to hospital. For example, it usually took 8–10 weeks in our institution. Thus, we collected data at different phases based on the radiotherapy timeline and conducted a Longitudinal analysis of the risk of RP ≥ 2 with RT for NSCLC of stage III/IV. Nevertheless, data regarding this topic remained missing. This work attempted to identify RP ≥ 2 risks as early as possible. With the introduction of data at different phases, the AUC values of the model gradually increased, eventually gaining a high AUC value of 0.9 in Phase I ~ III/I ~ IV models, higher than any single-phase model. Furthermore, the nomogram and risk classification system established based on the final model are helpful for individualized assessment of RP ≥ 2 risk and differentiation of RP ≥ 2 population. In the interpretation of our nomogram, we should consider internal validation from the statistical aspect. Apart from ROC survival analysis, this work performed DCA and calibration curves, and these were bootstrap validation approaches. As suggested by clinical statisticians, bootstrap validation is advantageous when the sample size is relatively small [49]. The internal validation results suggested that our nomogram had a satisfactory effect.

This study had several shortcomings, as follows. Firstly, it had a small sample size. More data are necessary for developing and validating our study. Moreover, this was a retrospective study, so there may be selection bias in our data. Third, at present, in our study, only common and important parameters in clinical practice were selected, including other valuable variables such as genomics and inflammatory markers that may be associated with RP. Therefore, we are also conducting studies in this area.

## Conclusion

In conclusion, the RP ≥ 2 risk study based on the radiotherapy timeline found that the RP ≥ 2 risk analysis model effect was more accurate as the radiotherapy process had advanced. Finally, we developed a new nomogram and risk classification system by including smoking status, radiomics feature of lungs, and lung and esophageal dose. Our results showed that the model has good performance, which can help doctors recognize high-risk RP ≥ 2 NSCLC cases of III/IV stage and guide personalized treatment and clinical decision-making.

## Data Availability

The full data and materials can be obtained from Shixiong Huang upon sufficient and reasonable request.

## Abbreviations

NSCLC: Non-small-cell lung cancer
RP: Radiation pneumonitis
RT: Radiation therapy
ROI: Region of interest
GTV: Gross tumor volume
CTV: Clinical target volume
PTV: Planning target volume
CSCO: Chinese Society of Clinical Oncology
NCCN: National Comprehensive Cancer Network
RP ≥ 2: Grade ≥ 2 Radiation pneumonitis
COPD: Chronic obstructive pulmonary disease
GLCM: Gray Level Concurrence Matrix
GLRLM: Gray Level Run Length Matrix
GLSZM: Gray Level Size Zone Matrix
GLDM: Gray Level Dependence Matrix
NGTDM: Neighbourhood gray-tone difference matrix
LASSO: Least absolute shrinkage and selection operator
ROC: Receiver operating characteristic
DCA: Decision curve analysis
PLT_3W: Platelet in the third week
OR: Odds ratio
95% CI: 95% confidence interval
auc: Area under curve
